# A case of progressive dyspnea: Atrial septal device-related functional mitral regurgitation after myocardial infarction

**DOI:** 10.34172/jcvtr.2021.04

**Published:** 2021-01-18

**Authors:** Kadriye Memic Sancar, Ayan Isık, Unal Aydın, Ali Kemal Kalkan, Mehmet Erturk, Gamze Babur Guler

**Affiliations:** ^1^Mehmet Akif Ersoy Thoracic and Cardiovascular Research and Education Hospital, Cardiology, Istanbul, Turkey; ^2^Mehmet Akif Ersoy Thoracic and Cardiovascular Research and Education Hospital, Cardiovascular Surgery, Istanbul, Turkey

**Keywords:** Atrial Septal Defect, Mitral Regurgitation, Device Closure

## Abstract

Transcatheter device closure is a common treatment option of atrial septal defect. Mitral regurgitation has been reported with comorbid mitral valve prolapse and atrial septal defect. However there is no consensus regarding the pathogenesis of mitral regurgitation after closure. We are reporting a patient with functional mitral regurgitation associated with both an oversize closure device and wall motion abnormality after inferior myocardial infarction.

## Introduction

Atrial septal defect (ASD) is a common congenital abnormality in both childhood and adult.^1^ Transcatheter device closure and surgical closure are both safely applied in the treatment of patients. After transcatheter ASD closure, sometimes the occurrence of mitral insufficiency or worsening of existing mitral insufficiency can be observed.^1^ The cause of this condition is not fully known. We presented the case with functional mitral regurgitation (MR) associated with the closure device, which emerged after inferior myocardial infarction (MI).

## Case Presentation

**Figure 1:**
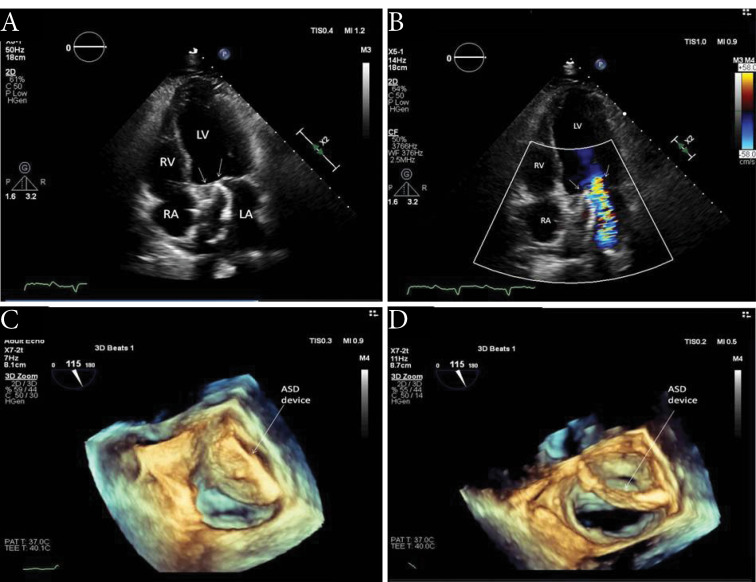
(A)Four chamber view of two-dimensional transthoracic echocardiography showing anterior leaflet compression related to oversized, malpozed device. LA, left atrium; LV, left ventricle; RA, right atrium; RV, right ventricle. (B) Two-dimensional transthoracic echocardiography with color Doppler; showing severe mitral regurgitation. Arrow indicated ASD occluder in between LA and RA septum. (C, D) Three dimensional echocardiography showing position of device. ASD, Atrial septal defect

**Figure 2:**
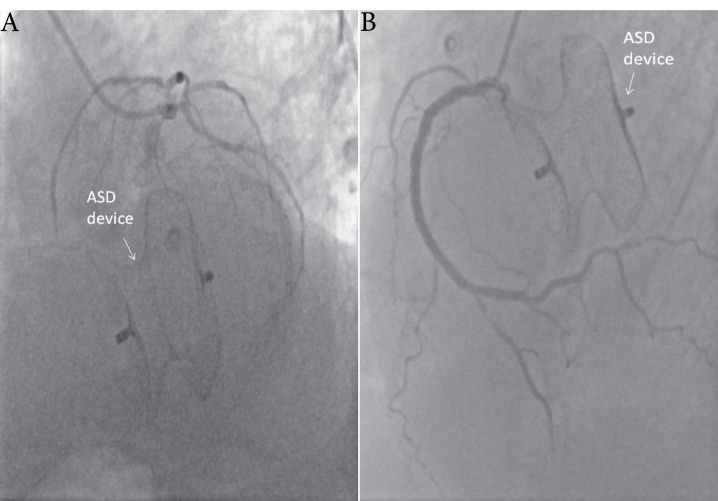
Left (A) and right (B) coronary angiogram showing oversized ASD occluder device. ASD, Atrial septal defect

**Figure 3:**
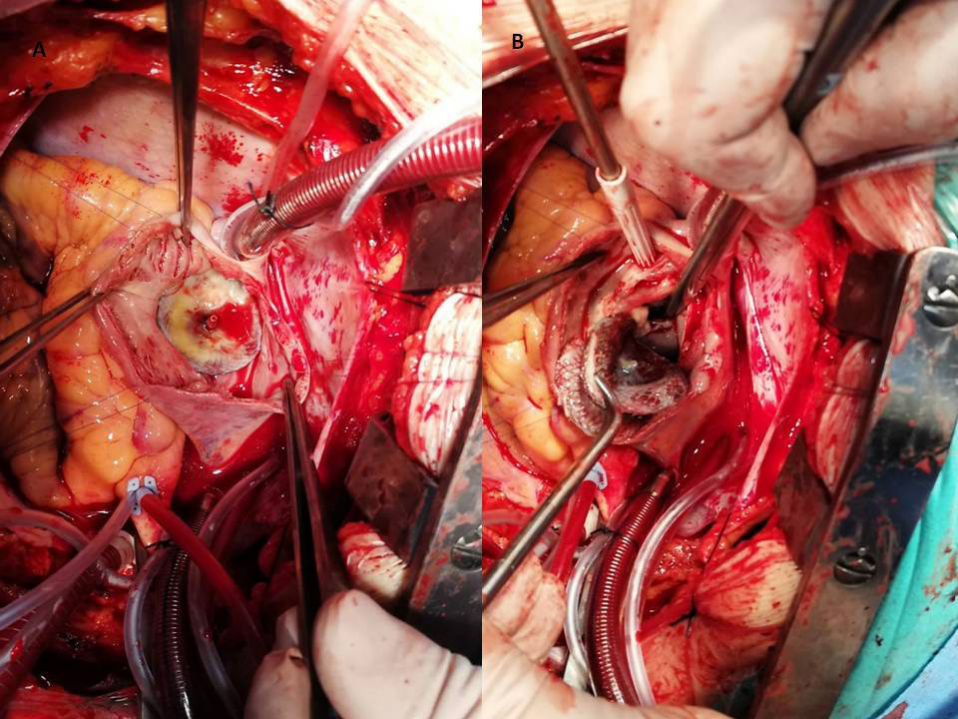
(A)Intraoperative view of device. (B) Surgeon’s comment: the device was intact but stuck to the septum and hardly seperated

A 56-year-old male patient presented with chest pain and progressive dyspnea for one year. The patient’s history revealed a percutaneous intervention with device closure of ASD seven years ago and acute inferior MI one year ago. He did not have any medical report about previous echocardiographic examinations. Physical examination on admission revealed a grade 4/6 apical systolic murmur, radiating to the axilla. His electrocardiography showed sinus tachycardia at 110 beats/min. The patient underwent transthoracic echocardiography and transesophageal echocardiography (TEE), documenting the presence of severe functional MR (regurgitant volume:61 ml, effective regurgitant orifice area: 0,4 cm^2^) with tethering at posterior leaflet related to basal and mid inferior wall akinesia and anterior leaflet compression related to device with reduced ejection fraction (EF 34 %) (Figure 1 A, B). The ASD device position and size were problematical in both two-dimensional (2D) and three-dimensional (3D) echocardiographic examination (Figure 1). The device was oversized, malposed and the margin of the device compressed the mitral anterior leaflet and posteromedial commissure (Figure 1). No shunt or perforation was detectable. A relative mitral stenosis (mean gradient 9 mmhg) releated to compression of mitral valve was observed. Coronary angiography showed two-vessel disease and ASD closure device malposition (Figure 2). The patient underwent surgical operation. Surgical inspection revealed that the device was intact but stuck to the septum and hardly seperated. It was oversized, malposed and ischemic macroscopic changes in both leaflet (more prominently in the posterior) was observed (Figure 3). No perforation also was observed in surgery. Following device removal, ASD closure with an autologous pericardial patch, mitral valve replacement combined with coronary artery bypass graft surgery were performed.

## Discussion

Atrial septal defect (ASD) is one of the most common adult congenital heart diseases.^2^ In the treatment of secundum ASD, transcatheter closure is a safe and comfortable method that is applied more often than surgical closure today. In percutaneous closure, anatomical compatibility, choosing the right patient and device are very important. The use of TEE and intracardiac echocardiography facilitated the use of these devices. Pre-operation TEE evaluation provides important information in measuring the size, location and number of ASD and evaluating the suitability for closure. 3D echocardiography also provides superiority over 2D echocardiography in determining the shape and number of ASD, while detecting the defect area; it also helps in determining the anatomy of the interatrial septum.^2^ An amount of percutaneous ASD closure contraindications have been reported to date; the diameter of the ASD defect is too large or too small (without transient ischemic attack or stroke history), insufficient rims, hemodynamic difficulties, complex anatomy, etc. ^3^ In our case, we could not discuss the indication of the closure because we do not have any clinical data before percutaneous closure. However, in echocardiographic examination, the device size was found to be larger than the septum and the dimensions of the right heart were normal values after closure.

Percutaneous ASD closure can also cause failures or complications, as with any procedure. The selection of a device that is not suitable for defect diameter, problems in rim measurement, mobile interatrial septum, technical problems and inexperience of the interventional team are among these reasons.^4^ In various publications, device embolization, complete AV block, myocardial erosion have been reported among these complications.^5,6^ In some patients, worsening of heart failure, atrial arrhythmia^7,8^ and deterioration of MR after closure may be observed.^9,10^ In a recent trial, Nishimura et al, reported that mitral anular dilatation associated with atrial fibrillation and a short posterior leaflet with a thick anterior leaflet are independent predictors of worsening MR after surgical ASD closure.^11^ The mechanisms of these adverse developments remain unclear.^12^ ASD closure is expected to decrease the volume load of the right ventricle and normalize the heart structure as a result of the elimination of the left-right shunt, it is speculated that this will alter the structure of the mitral annulus. ^13^

In the present case, it is thought that the changes in left ventricle geometry secondary to inferior MI has affected the configuration of the mitral annulus. However, huge device compressing the anterior leaflet and restriction of posterior leaflet secondary to low EF might be associated with the aggravation of preexisting MR or new-onset severe MR.

## Conclusion

In conclusion, to our knowledge this is the first case ever reported of functional mitral regurgitation caused by an oversized atrial septal occluder and abnormal left ventricular-mitral annular geometry. The importance of imaging during device selection and placement is once again understood for an oversized and malposed device. It is also important to note that regular postoperative follow-up of patients who have undergone transcatheter ASD closure may provide a chance for early intervention of complications.

## Competing interests

The authors report no conflict of interest. The authors alone are responsible for the content and writing of paper.

## Ethical approval

Informed consent has been obtained from the patient to publish this material.

## References

[1] Yang MC, Wu JR (2018). Recent review of transcatheter closure of atrial septal defect. Kaohsiung J Med Sci.

[2] Connolly HM, Taggart N. Surgical and percutaneous closure of atrial septal defects in adults. In: UpToDate, Post, TW (Ed). Waltham, MA: UpToDate; 2014.

[3] Fraisse A, Latchman M, Sharma SR, Bayburt S, Amedro P, di Salvo G (2018). Atrial septal defect closure: indications and contra-indications. J Thorac Dis.

[4] Chiappini B, Gregorini R, Di Eusanio M, Ciocca M, Villani C, Minuti U (2008). Embolization of an Amplatzer atrial septal closure device to the pulmonary artery. J Card Surg.

[5] Moore J, Hegde S, El-Said H, Beekman R 3rd, Benson L, Bergersen L (2013). Transcatheter device closure of atrial septal defects: a safety review. JACC Cardiovasc Interv.

[6] Chessa M, Carminati M, Butera G, Bini RM, Drago M, Rosti L (2002). Early and late complications associated with transcatheter occlusion of secundum atrial septal defect. J Am Coll Cardiol.

[7] Ewert P, Berger F, Nagdyman N, Kretschmar O, Dittrich S, Abdul-Khaliq H (2001). Masked left ventricular restriction in elderly patients with atrial septal defects: a contraindication for closure?. Catheter Cardiovasc Interv.

[8] Masutani S, Senzaki H (2011). Left ventricular function in adult patients with atrial septal defect: implication for development of heart failure after transcatheter closure. J Card Fail.

[9] Joy J, Kartha CC, Balakrishnan KG (1993). Structural basis for mitral valve dysfunction associated with ostium secundum atrial septal defects. Cardiology.

[10] Suchoń E, Podolec P, Płazak W, Tomkiewicz-Pajak L, Pieculewicz M, Mura A (2004). Mitral valve prolapse associated with ostium secundum atrial septal defect--a functional disorder. Acta Cardiol.

[11] Nishimura S, Izumi C, Amano M, Miyake M, Tamura T, Kondo H (2017). Incidence and predictors of aggravation of mitral regurgitation after atrial septal defect closure. Ann Thorac Surg.

[12] Nagata S, Nimura Y, Sakakibara H, Beppu S, Park YD, Kawazoe K (1983). Mitral valve lesion associated with secundum atrial septal defect Analysis by real time two dimensional echocardiography. Br Heart J.

[13] Hiraishi M, Tanaka H, Motoji Y, Sawa T, Tsuji T, Miyoshi T (2015). Impact of right ventricular geometry on mitral regurgitation after transcatheter closure of atrial septal defect. Int Heart J.

